# mfapy: An open-source Python package for ^13^C-based metabolic flux analysis

**DOI:** 10.1016/j.mec.2021.e00177

**Published:** 2021-07-17

**Authors:** Fumio Matsuda, Kousuke Maeda, Takeo Taniguchi, Yuya Kondo, Futa Yatabe, Nobuyuki Okahashi, Hiroshi Shimizu

**Affiliations:** Department of Bioinformatic Engineering, Graduate School of Information Science and Technology, Osaka University, 1-5 Yamadaoka, Suita, Osaka, 565-0871, Japan

**Keywords:** ^13^C-based metabolic flux analysis, Experimental design, Non-linear optimization, Open-source software, Python package

## Abstract

^13^C-based metabolic flux analysis (^13^C-MFA) is an essential tool for estimating intracellular metabolic flux levels in metabolic engineering and biology. In ^13^C-MFA, a metabolic flux distribution that explains the observed isotope labeling data was computationally estimated using a non-linear optimization method. Herein, we report the development of mfapy, an open-source Python package developed for more flexibility and extensibility for ^13^C-MFA. mfapy compels users to write a customized Python code by describing each step in the data analysis procedures of the isotope labeling experiments. The flexibility and extensibility provided by mfapy can support trial-and-error performance in the routine estimation of metabolic flux distributions, experimental design by computer simulations of ^13^C-MFA experiments, and development of new data analysis techniques for stable isotope labeling experiments. mfapy is available to the public from the Github repository (https://github.com/fumiomatsuda/mfapy).

## Highlights

• An open-source Python package, mfapy, is developed for ^13^C-MFA.
• mfapy enables users to write Python codes for data analysis procedures of ^13^C-MFA.
• mfapy has a flexibility and extensibility to support various data analysis procedures.
• Computer simulations of ^13^C-MFA experiments is supported for experimental design.

## 1 Introduction

Measurement of intracellular metabolic flux is essential for metabolic engineering and biology (Antoniewicz, 2013, Antoniewicz, 2015; Wiechert, 2001; Wittmann, 2007; Zamboni et al., 2009). ^13^C-based metabolic flux analysis (^13^C-MFA) was developed to estimate metabolic flux levels in the central carbon metabolism of metabolically engineered microbial cells (Costenoble et al., 2007; Shirai et al., 2007; Wasylenko and Stephanopoulos, 2015). Recently, it has been applied to the quantitative analysis of cell metabolism in plants, mammalian cells, and cancers (Christen and Sauer, 2011; Gaglio et al., 2011; Haverkorn van Rijsewijk et al., 2011; Hiller and Metallo, 2013; Shimizu, 2004).

To perform ^13^C-MFA, cells are cultivated in a medium containing ^13^C-labeled carbon sources (Antoniewicz, 2018; Cheah et al., 2017; McAtee Pereira et al., 2018). Amino acids or intermediates are extracted and subjected to mass spectrometric analysis to measure the isotopic labeling enrichment or mass isotope distribution vector (MDV) of each metabolite. Additionally, specific rates for carbon source consumption and product excretion are determined from culture profile data. Because the MDV reflects intracellular metabolic flux levels, a metabolic flux distribution that effectively explains the observed data is computationally estimated using a non-linear optimization method (Matsuda et al., 2017). Several software packages can be used to perform the data analysis, such as 13CFLUX2 (Weitzel et al., 2013), C13 (Cvijovic et al., 2010), Metran (Yoo et al., 2008), INCA (Young, 2014), influx_s (Sokol et al., 2012), OpenFLUX2 (Shupletsov et al., 2014), WUflux (He et al., 2016), FluxPyt (Desai and Srivastava, 2018), and OpenMebius (Kajihata et al., 2014). These software packages contain functions required for the modern ^13^C-based MFA, including the rapid calculation of isotopic labeling enrichment using the elementary metabolite unit (EMU) framework (Antoniewicz et al., 2007b), determination of the confidence interval of the estimated flux level (Antoniewicz et al., 2006), parallel labeling experiments (Ahn and Antoniewicz, 2013; Crown et al., 2015; Leighty and Antoniewicz, 2013), and isotopically non-stationary MFA (INST-MFA) (Jazmin et al., 2014; Schaub et al., 2008; Young et al., 2008, 2011). Useful command-line-based or user-friendly graphic interfaces have been prepared for routine ^13^C-MFA. Moreover, several software packages have been distributed with open-source licenses (Desai and Srivastava, 2018; Kajihata et al., 2014; Sokol et al., 2012).

This article describes the development of mfapy, an open-source Python package for data processing of ^13^C-based metabolic flux analysis. Previously, we reported on OpenMebius for use in MATLAB (Kajihata et al., 2014). The advantages of MATLAB are its fast and reliable calculation environment with the manufacturer's support and many users, especially in chemical engineering field. However, Python was employed for the development of mfapy because Python is a freely available, popular programing language recently used for scientific purposes. A possible disadvantage is that Python is considered to be slow for operations on numerical data.

A novelty of mfapy is that it provides a set of functions required for a^13^C-based metabolic flux analysis as a Python package. In OpenMebius, a metabolic flux distribution can be estimated by six commands and Excel files, including model definition and experimental data. The command-line-based interface is useful for routine data processing of ^13^C-MFA. However, mfapy employs an object-oriented style interface that provides more than 70 methods to manipulate data and metabolic models for ^13^C-MFA. The flexibility and extensibility allow users to write or customize Python codes to describe various procedures for data analysis in isotope labeling experiments. For instance, computer estimation of the metabolic flux distribution by non-linear optimization requires trial and error to obtain the global optimum result without being trapped in local optima. Some flexibility is required in the routine data processing of ^13^C-MFA, such as selecting suitable non-linear optimization solvers to identify a plausible candidate for the global optimum. Moreover, a useful ^13^C-labeled carbon source must be selected to design a practical isotopic labeling experiment. Hence, a computer simulation of the isotopic labeling experiment was performed using artificial MDV data (Maeda et al., 2016). Furthermore, extensibility is required to develop advanced data analysis techniques, such as Monte Carlo-based algorithms, to generate a probability distribution of metabolic flux levels.

This study demonstrates that the flexibility and extensibility provided by mfapy enable us to write Python scripts for trial and error in routine ^13^C-MFA tasks, for computer simulation of ^13^C-MFA experiments to select suitable carbon sources, and for the development of new data analysis techniques. This article introduces various examples of Python scripts describing the procedure of ^13^C-MFA and other workflows. All Python scripts and related files mentioned in this study can be downloaded from the GitHub repository (https://github.com/fumiomatsuda/mfapy).

## 2 Materials and Methods

### 2.1 Implementation of mfapy and code availability

**Fig. 1:**
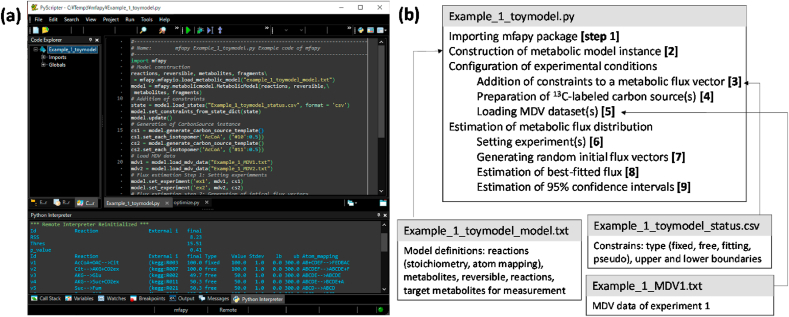
Procedure for ^13^C-MFA by mfapy. (a) Screenshot of a python environment (PyScripter) running mfapy. (b) A typical scheme for estimating a best fitted metabolic flux distribution using the model definition, status, and MDV data files. Python codes for the parallel labeling experiment and the metabolic network of the toy model are shown in Supplementary Figs. 1 and 2.

mfapy is implemented in Python 3 based on external packages, including NumPy (Harris et al., 2020), SciPy (Virtanen et al., 2020), nlopt (the non-linear optimization package by Steven G. Johnson, http://github.com/stevengj/nlopt), joblib (https://joblib.readthedocs.io/en/latest/), and mkl-service (Fig. 1a). We tested all mfapy functions using the 64-bit version of Anaconda3 (https://anaconda.com) for Windows. All scripts and example files of mfapy, detailed explanations of all functions, rules that describe the user-defined metabolic pathway, and the carbon transition network are available on the project home page (https://github.com/fumiomatsuda/mfapy). Documentation of each mfapy function is also available from GitHub (https://fumiomatsuda.github.io/mfapy-document/).

The software comprises five modules: mfapy.mfapyio, mfapy.metabolicmodel, mfapy.mdv, mfapy.carbonsource, and mfapy.optimize. The mfapy.optimize submodule includes low-level functions for various optimization tasks and is not used by users directly. Fig. 1b shows a typical scheme for estimating the best-fitted metabolic flux distribution using mfapy. The mfapy package must be imported at the beginning of the Python 3 scripts (Fig. 1b, step 1). The corresponding Python code for the ^13^C-MFA of the toy model is shown in Supplementary Fig. 1.

### 2.2 Model construction

In ^13^C-MFA, a metabolic model *M* is constructed based on a metabolic pathway network and carbon transition network. *M* is a function for calculating the isotopic labeling enrichment or a mass isotope distribution vector (*MDV*^*sim*^) of metabolites from a specified vector of metabolic flux (*v*) and isotopic labeling patterns of carbon sources (*x*^*inp*^).

(1) $$MDV_{j}^{\;sim}\;=\;M\left(v,\;x^{inp}\right)$$

Because the metabolic flux distribution at a metabolically steady state is determined, the vector of metabolic flux (*v*) follows the stoichiometric equation, *Sv* = 0, where *S* is the stoichiometric matrix produced from the stoichiometry of metabolic reactions. $MDV_{j}^{\;sim}$ is calculated by the framework of EMUs (Antoniewicz et al., 2007b) using the carbon transition information of each metabolic reaction. In mfapy, the metabolic model *M* is implemented as a MetabolicModel class in the mfapy.metabolicmodel module. An instance of the MetabolicModel class can be generated from a model definition file describing the stoichiometry and carbon transition information of each metabolic reaction (Fig. 1b, step 2, Table 1). The number of carbons, symmetry, and other metabolite properties as well as reversible reactions and target fragments for measurements are also described in the model definition file (Table 1 and Supplementary Table 1).

**Table 1:** Example of model definition file (extracted from Supplementary Table 1 or Example_1_toymodel_model.txt).

//Reactions				
Id	For Stoichiometry Matrix	For Atom mapping	Atom mapping	Link to external ID
v1	AcCoA + OAC-- > Cit	AcCoA + OAC-- > Cit	AB + CDEF-- > FEDBAC	(kegg:R00351)
v2	Cit-- > AKG + CO2ex	Cit-- > AKG + CO2ex	ABCDEF-- > ABCDE + F	(kegg:R00709)
v3	AKG-- > Glu	AKG-- > Glu	ABCDE-- > ABCDE	(kegg:R00243)
v4	AKG-- > Suc + CO2ex	AKG-- > Suc + CO2ex	ABCDE-- > BCDE + A	(kegg:R01197)
v5	Suc-- > Fum	Suc-- > Fum	ABCD-- > ABCD	(kegg:R02164)
v6	Fum-- > OAC	Fum-- > OAC	ABCD-- > ABCD	(kegg:R01082)
v7	OAC-- > Fum	OAC-- > Fum	ABCD-- > ABCD	(kegg:R01082)
v8	Asp-- > OAC	Asp-- > OAC	ABCD-- > ABCD	(kegg:R00355)
v9	Glu-- > Gluex	nd	nd	(kegg:R00243)
//Metabolites				
Name	Number of carbons	Symmetry	Carbon source	Excreted metabolite
CO2ex	1	no	no	excreted
AcCoA	2	no	carbonsource	no
OAC	4	no	no	no
Cit	6	no	no	no
AKG	5	no	no	no
Suc	4	symmetry	no	no
Fum	4	symmetry	no	no
Glu	5	no	no	no
Gluex	5	no	no	excreted
Asp	4	no	carbonsource	no

### 2.3 Addition of constraints

mfapy considers four types of metabolic reactions, including “free,” “fixed,” “fitting,” and “pseudo” types, to constrain *v* by observed rates of biomass synthesis, substrate consumption, and product excretion. The metabolic flux level of a “free” reaction is a free variable between the lower and upper boundaries. The metabolic flux level of a “fixed” reaction is constrained to a specified observed flux value. The metabolic flux level of a “fitting” reaction is a variable between the lower and upper boundaries, and its residue against a specified flux value is considered in the residual sum of squares (RSS) in the model fitting. The “pseudo”-type reactions are disregarded in constructing a stoichiometry matrix of its substrate (Supplementary Fig. 3) (Desai and Srivastava, 2018). These constraints for each reaction are described in “Example_1_toymodel_status.csv” (Table 2) and applied to the model (Fig. 1b, step 3).

**Table 2:** Example of a status file (Example_1_toymodel_status.csv).

Class	Id	type	Value^1)^	Standard deviation^2)^	Lower boundary	Upper boundary
reaction	v1	fixed	100	1	0	300
reaction	v2	free	100	1	0	300
reaction	v3	free	50	1	0	300
reaction	v4	free	50	1	0	300
reaction	v5	free	50	1	0	300
reaction	v6	free	125	1	0	300
reaction	v7	free	75	1	0	300
reaction	v8	free	50	1	0	300
reaction	v9	free	50	1	0	300
reversible	FUM	free	50	1	0	300

1) Metabolic flux level information was used for fixed and fitting types.

2) Standard deviation information was used for fitting type only.

### 2.4 Labeling patterns of carbon sources

mfapy comprises a CarbonSource class to handle the isotopic labeling information of carbon sources (*x*^*inp*^). An instance of the class is automatically generated from a MetabolicModel instance by the “generate_carbon_source_template” method. The isotope labeling patterns can be set using the “set_each_isotopomer” method (Fig. 1b, step 4).

### 2.5 Loading of observed MDV data

The isotopic labeling enrichment of metabolites is described by the MDV (Wittmann and Heinzle, 1999):

(2) $$MDV_{j}=\left[\begin{array}{l} \begin{array}{l} m+0\\ m+1 \end{array}\\ \vdots \\ m+n \end{array}\right]\;with\;\sum_{i\;=\;0}^{n}m+i\;=\;1$$

where *MDV*_*j*_ is the vector of isotopic labeling enrichment of metabolite *j*;m+i indicates the relative abundance of a metabolite, in which *i* carbons are labeled with ^13^C. The mass spectrum data are rectified for the presence of naturally occurring isotopes using a correction matrix to obtain the *MDV*_*j*_ of the carbon skeleton (van Winden et al., 2002). An observed MDV dataset described in “Example_1_MDV1.txt” yields an instance of the MdvData class (Table 3 and Fig. 1b, step 5).

**Table 3:** Example of MDV file (Example_1_MDV1.txt).

Name	Isotopomer	Select	MDV	Standard deviation
GluMeas	0	1	0.012477	0.01
GluMeas	1	1	0.733379	0.01
GluMeas	2	1	0.254143	0.01
GluMeas	3	1	0.0	0.01
GluMeas	4	1	0.0	0.01
GluMeas	5	1	0.0	0.01

### 2.6 Metabolic flux estimation

A vector of metabolic flux *v* is fitted to the observed mass spectrum ($M\hat{D}V_{j}$) using a non-linear optimization method:

$$RSS\left(v\right)\;=\;\overset{N}{\underset{j\;=\;1}{\sum }}\left({\left[M\hat{D}V_{j}\;-\;MDV_{j}^{\;sim}\right]}^{T}C_{M\hat{D}V_{j}}^{\;-1}\left[M\hat{D}V_{j}\;-\;MDV_{j}^{\;sim}\right]\right)$$

(3) $$\begin{array}{l} v_{opt}=\arg\min_{v}RSS\left(v\right)\\ \;s.t.\;Sv=0 \end{array}$$

The optimized value $v_{opt}$ is the estimated metabolic flux distribution in the cells to minimize the covariance-weighted sum of squared difference. $C_{M\hat{D}V_{j}}$is the covariance matrix with a measurement standard deviation located on the diagonal.

In mfapy, the estimation of $v_{opt}$ is executed by a three-step procedure using the methods of the MetabolicModel class. In step 6 in Fig. 1b, a pair of observed MDV datasets and the isotopic labeling information of carbon sources are registered as “a labeling experiment” using the set_experiment method. In step 7 in Fig. 1b, a random initial flux distribution is generated using the generate_initial_states method. In step 8 in Fig. 1b, the metabolic flux vector *v* is optimized using the fitting_flux method to minimize the covariance-weighted sum of squared difference (Eq. [3]). To solve the non-linear optimization problem, mfapy employs Scipy and nlopt packages supporting 13 global and local optimizers, such as sequential quadratic programming (SLSQP) and CRS2_LM (“controlled random search with local mutation”).

### 2.7 Determination of confidence intervals

The confidence intervals of the estimated fluxes are determined using the grid search method (Antoniewicz et al., 2006; Costenoble et al., 2007). Following the generation of a template dictionary (ci_enge) to describe the target reactions, the 95% confidence interval is determined using the search_ci method (Fig. 1b, step 9).

### 2.8 Generation of artificial MDV

An artificial observed MDV is used for computer simulation of the ^13^C-MFA experiment. For a specified metabolic flux vector, *v*, and carbon source (such as the cs prepared above), an MDVdata instance, including simulated MDV data, can be generated by the “generate_mdv” method of the MetabolicModel instance. The MDVdata instance comprises “add_gaussian_noise” and “set_std” to add Gaussian noise to the MDV data and set standard deviation levels for the MDV measurement. A Python code example for this task is as follows:

#Generation of artificial MDV

mdv = model.generate_mdv(v, cs)

mdv.add_gaussian_noise(0.01)

mdv.set_std(0.01)

### 2.9 Metropolis-Hastings algorithm

The Metropolis-Hastings algorithm is performed as follows:

(1) A seed metabolic flux distribution *v* is produced by minimizing the RSS(*v*) using the MetabolicModel.fitting_flux method, as described above.
(2) Based on the flux distribution at the j^th^ step, *v*, a proposal flux distribution, *v’*, is generated by adding random values to the flux level of three randomly selected reactions. Proposal flux distributions are iteratively generated until a *v’* within a feasible flux space is obtained using the MetabolicModel.add_perturbation method.
(3) If the acceptance probability *p* = *P* (RSS(*v’*))/*P* (RSS(*v*)) is larger than 1.0*,* then (*v’*) is accepted for the next step. *P* is the probability distribution of the χ^2^ distribution (the degree of freedom is the number of measurements). If *p* < 1.0, *v’* is accepted with probability *p.* When *v’* is rejected, *v* is used in the next step.
(4) The procedure is repeated 5,000,000 times to generate a Markov chain because at least 100,000–1,000,000 steps are required to obtain a stable estimate (Matsuda et al., 2020). The data of the initial 2,500,000 steps are discarded during the burn-in process.
(5) The remaining chain with 2,500,000 steps is used as the sample population of *v*, following a posterior distribution. From the population, 2500 data points are obtained by sampling every 1000 steps of the chain.
(6) The entire procedure is performed 20 times via parallel computing.
(7) A sample population of 50,000 data points (2500 with 20 replicates) is used for the following data analysis. The entire procedure is available in the MetabolicModel.posterior distribution method.

### 2.10 Test data

A toy model of the tricarboxylic acid (TCA) cycle was obtained from a previous study (Antoniewicz et al., 2007b). Two ^13^C-MFA datasets described in our previous study, including a metabolically engineered *Escherichia coli* strain producing isopropanol (Okahashi et al., 2017) and breast cancer cells (Araki et al., 2018), were used as examples.

## 3 Results

### 3.1 Test of mfapy: MDV calculation using an EMU algorithm

mfapy uses the framework of the EMU algorithm to determine a simulated MDV ($MDV_{j}^{\;sim}$) from vectors of the metabolic flux (*v*) and the isotopic labeling pattern of the carbon source (*x*^*inp*^) (Antoniewicz et al., 2007b). In the original article about the EMU algorithm, an example of MDV calculation based on a toy model of the TCA cycle, including nine reactions and 10 metabolites, was introduced (Supplementary Fig. 2) (Antoniewicz et al., 2007b). In this study, we prepared a model definition file of the toy model (Example_0_toymodel_model.txt, Supplementary Table 1) and a Python script describing a procedure to calculate the MDV of glutamate (Example_0_toymodel.py). In the procedure, following the construction of the metabolic model from the model definition file, the MDV of glutamate ($MDV_{Glu}^{\;sim}$) was determined using Eq. (1) using *v* and *x*^*inp*^ from the original literature (100% non-labeled Asp and [non-labeled AcCoA: [1–^13^C]Ac-CoA: [U–^13^C]Ac-CoA] = [50:25:25]). The obtained $MDV_{Glu}^{\;sim}$ was identical to the theoretical values shown in the original literature (Supplementary Table 2), indicating that the EMU algorithm was implemented exactly and performed as intended in mfapy.

### 3.2 Test of mfapy: ^13^C-MFA of metabolically engineered *E. coli*

In our previous study, ^13^C-MFA was conducted to estimate the metabolic flux distribution of the central carbon metabolism of a metabolically engineered *E. coli* strain to produce isopropanol using the authentic ^13^C-MFA software OpenMebius (Okahashi et al., 2017). The metabolically engineered strain (MSI002 strain) was cultured in an M9 medium containing ^13^C-labeled glucose (glucose : [1–^13^C]glucose : [U–^13^C]glucose = 2:70:28). Cells were collected in an exponential growth phase (OD600 ~1) to measure the MDV of proteinogenic amino acids by GC-MS. Furthermore, the specific rates for glucose consumption, and acetate, acetone, and isopropanol production were determined from the culture profile data. The metabolic flux distribution of central carbon metabolism was successfully estimated from the MDV of 23 fragments of proteinogenic amino acids and specific rate data using the metabolic model of *E. coli*, including 85 reactions. G-value parameters were included in each target fragment by applying a patch to the source code of OpenMebius to rectify the effects of inoculated unlabeled proteinogenic amino acids on the observed MDV (Antoniewicz et al., 2007a; Okahashi et al., 2017).

Here, the procedure for data processing in the previous ^13^C-MFA study was transported to a Python code using mfapy functions (“Example_2_1_Ecoli.py”). A metabolic model definition file (“Example_2_Ecoli_model.txt”) and status file (“Example_2_Ecoli_status.csv”) were prepared from the original data from the literature. To manage the G-value parameters, a specific reaction type, “pseudo,” can be used in mfapy (see Supplementary Fig. 3 for details). The metabolic flux vector *v* was optimized to minimize the difference between the observed and simulated MDVs (Eq. [3]). The metabolic flux vector *v* of the best-fitted optimization determined via mfapy was identical to that determined using OpenMebius in a previous study (Supplementary Fig. 4, Supplementary Table 3). This task required approximately 0.4 h when performed using AMD Ryzen 9 3900X (12 core, 3.8G Hz) processor.

### 3.3 Function of mfapy: availability of multiple solvers

mfapy employs Scipy and nlopt external packages to support 13 global and local non-linear optimization solvers. Various solvers with distinct algorithms have been developed owing to the compatibility between the algorithms and non-linear optimization problems. To investigate the compatibility with ^13^C-MFA, the performance of 13 solvers was compared by solving the *E. coli* example (Example_2_2_Ecoli_solver_comparison.py).

**Fig. 2:**
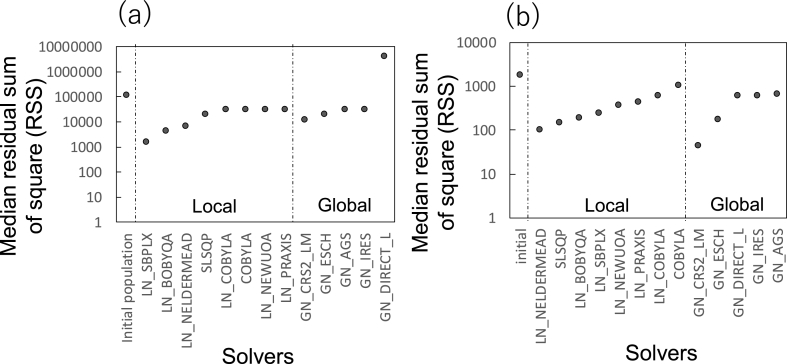
Performance comparison of 13 non-linear optimization solvers. Following the preparation of 100 initial metabolic flux vectors, each metabolic flux vector was optimized for 60 s using a non-linear optimization solver. Median RSSs of 100 metabolic flux vectors are shown in the figure. (a) Comparison using a^13^C-MFA dataset of *E. coli* (Okahashi et al., 2017). (b) Comparison using a^13^C-MFA dataset of cancer cells (Araki et al., 2018).

For example, following a generation of a population of 100 random initial metabolic flux vectors, each metabolic flux vector was optimized for 60 s using the SLSQP algorithm. The median RSS level among the 100 metabolic flux vectors was 20,896 (Fig. 2a). The procedure was repeated for the 13 solvers using an identical population of the initial metabolic flux vectors. Fig. 2a shows that, among the eight local optimization solvers, the best result was obtained for “LN_SBPLX (Subplex),” whose median RSS value was 1660. The second-best solver was “LN_BOBYQA” (bound optimization by quadratic approximation; median RSS value of 4400). For the cases of global solvers, the best result was obtained for “GN_CRS2_LM (controlled random search with local mutation)” with a median RSS value of 12,953.

A similar comparison was performed using a dataset obtained from a previous ^13^C-MFA study of breast cancer (MCF-7) cells (Araki et al., 2018) (Example_3_MCF7.py). MCF-7 breast cancer cells were cultured in media containing non-labeled glucose and [U–^13^C]glutamine, as well as [1–^13^C]glucose and non-labeled glutamine, in parallel. Intracellular metabolites were extracted at 24 h and analyzed via mass spectrometry to obtain the MDV of 16 fragments of 7 metabolites. The specific rates for glucose, glutamine consumption, and lactate production were determined from the culture profile data. The MDV, specific rate data, and the metabolic model for MCF-7 including 85 reactions obtained in the previous study were used for the comparison of 13 optimization solvers in this study.

The result showed that “GN_CRS2_LM” yielded the best median RSS value of 44 among five global solvers, although performance of other global optimization solvers depended heavily on the dataset (Fig. 2b). Furthermore, the best and second-best local solvers were “LN_NELDERMEAD (the Nelder–Mead simplex algorithm)” and “SLSQP,” with median RSS values of 103 and 150, respectively. For local optimization, “LN_SBPLX (Subplex),” “LN_BOBYQA,” “LN_NELDERMEAD,” and “SLSQP” were typically better than the other solvers in the two examples. We empirically selected “GN_CRS2_LM” and “SLSQP” as the first option in a new ^13^C-MFA study, and tested other solvers because of the solver dependency on datasets.

### 3.4 Function of mfapy: parallel execution of optimization tasks for finding the global optimum

Non-linear optimization solvers often fail to reach the global optimum because they are trapped in a local optimum or their optimization progress is slow. To avoid local optima, mfapy can execute optimization trials from many random initial metabolic flux vectors in parallel processors using a task-parallel execution environment of the joblib module. In this study, global and local optima were investigated using the *E. coli* example above. The procedure for model fitting was changed by modifying the Python code (Example_2_3_Ecoli_local_optimum.py). An optimization trial comprised the global optimization by GN_CRS2_LM with 10,000 optimization steps, followed by a gradient-based local optimization (SLSQP). Optimization trials from 1000 random initial metabolic flux vectors were executed in parallel, while the optimization progression was monitored.

**Fig. 3:**
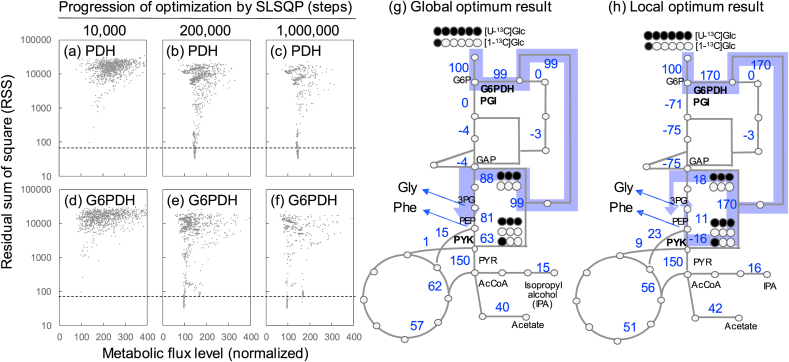
Investigation of global and local optima in ^13^C-MFA of metabolically engineered *E. coli*. Metabolic models and measurement data were obtained from a previous study (Okahashi et al., 2017). Model fitting was performed by the gradient-based local optimization (SLSQP). A total of 1000 optimization trials were executed in parallel. (a–f) Progression of optimization of 1000 trials. Metabolic flux levels of PDH (a–c) and G6PDH (d–f) reactions at the 10,000^th^ (a and d), 200,000^th^ (b and e), and 1,000,000th (c and f) steps are shown in figure. (g and h) Metabolic flux distribution of global (g) and local (h) optimum results. Blue numbers represent metabolic flux levels. Blue lines indicate significant carbon flow toward glycine and phenylalanine. White and black circles indicate ^13^C-labeling patterns of carbon source (glucose, Glc) and GAP and PYR produced by the ED pathway. All metabolic flux levels are normalized to that of the glucose uptake rate. (For interpretation of the references to colour in this figure legend, the reader is referred to the Web version of this article.)

A comparison between the RSS and metabolic flux level of pyruvate dehydrogenase (PDH) showed that optimizations remained incomplete after the 10,000 steps of SLSQP (Fig. 3a). The RSS of 106 and 359 trials were below the threshold after 200,000 and 1,000,000 SLSQP steps, respectively (Fig. 3b and c). The metabolic flux level of PDH converged to a single value as optimization progressed (Fig. 3c).

Furthermore, a comparison between the RSS and metabolic flux level of the reaction of glucose 6-phosphate dehydrogenase (G6PDH) revealed a global and local optimum below the RSS threshold. Although 339 trials reached the global optimum, 20 trials were trapped in the local minimum after 1,000,000 steps (Fig. 3d–f).

A metabolically engineered strain (MSI002 strain) was constructed in a previous study by removing the phosphoglucose isomerase (PGI) reaction and activation of the Entner–Doudoroff (ED) pathway (Okahashi et al., 2017). However, ^13^C-MFA was conducted using a metabolic network, including a reversible PGI reaction. Moreover, the reactions of fructose 1,6-bisphosphatase (FBPase; FBP→F6P) and PEP synthetase (Pps; pyruvate→PEP) were considered based on experimental validation (Okahashi et al., 2017). The metabolic flux vector of the global optimum result showed that the metabolic flux level of the PGI reaction was successfully determined to be zero (Fig. 3g, Supplementary Table 3). Glucose was catabolized via the ED pathway to produce glyceraldehyde 3-phosphate (GAP) and pyruvate (PYR). Subsequently, GAP was converted to PYR by the lower Embden–Meyerhof–Parnas (EMP) pathway (Fig. 3g).

In the local optimum, the GAP produced by the ED pathway was converted to glucose 6-phosphate (G6P) via the gluconeogenesis pathway and entered the ED pathway again, as shown in Fig. 3h. A comparison between the observed MDV and the simulated MDVs of the global and local optima suggested that the RSS of glycine-derived fragments in the local optimum was smaller than that of the global optimum (Supplementary Table 4). In contrast, the RSS of the m+3 signals of phenylalanine-related fragments significantly increased in the local optimum. This implies that, in the global optimum result, the [U–^13^C]GAP and non-labeled GAP produced via the ED pathway were primarily used for glycine and phenylalanine biosynthesis because of the high metabolic flux levels of the lower EMP pathway (Fig. 3g). In contrast, for the local optimum results (Fig. 3h), [U–^13^C]Pyr, [1–^13^C]Pyr and non-labeled Pyr produced via the ED pathway were used for the synthesis of glycine and phenylalanine. An optimization descent may be trapped in the local minimum when a metabolic flux vector *v* possesses the latter distribution during the optimization process. The results confirmed that the local optimum was caused by the characteristics of the metabolic network and the small experimental error in the observed MDV of glycine. Furthermore, the example showed that many optimization trials are required to obtain the global optimum.

### 3.5 Function of mfapy: generation of artificial observed MDV for simulating ^13^C-MFA experiments

In the ^13^C-MFA, 95% confidence intervals were determined for each metabolic reaction to evaluate the reliability of the flux estimation. To minimize the 95% confidence intervals, a suitable ^13^C-labeled carbon source should be used for labeling experiments (Crown and Antoniewicz, 2012). A computer simulation of a^13^C-MFA experiment is useful in the determination of suitable carbon sources (Maeda et al., 2016). mfapy includes required functions, such as the generation of an artificial observed MDV (see Materials and Methods).

**Fig. 4:**
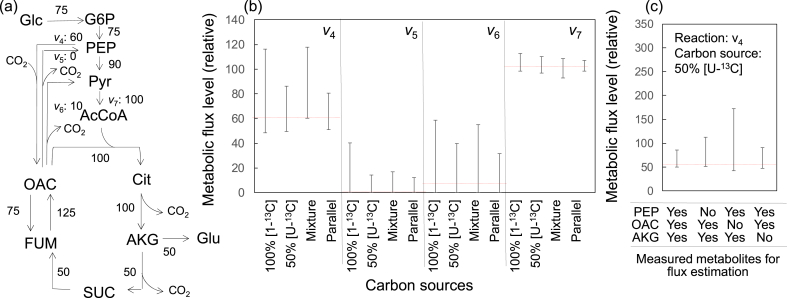
Computer simulation of ^13^C-MFA for the selection of an effective carbon source. (a) Expanded toy model of central carbon metabolism of *E.coli*. (b) Comparison of 95% confidence intervals of three anaplerotic reactions (*v*_4_, *v*_5_, and *v*_6_) and one non-anaplerotic reaction (*v*_7_) determined via simulation of ^13^C-MFA using distinct carbon sources. Red dotted lines represent metabolic flux levels of answers. [1–^13^C]glucose: 100% [1–^13^C]glucose, [U–^13^C]glucose: [U–^13^C]glucose and non-labeled glucose at 50:50 ratio, Mixture: [U–^13^C]glucose and [1–^13^C]glucose at 50:50 ratio, Parallel: parallel labeling experiment using 100% [1–^13^C]glucose and the mixture of [U–^13^C]glucose and non-labeled glucose at 50:50 ratio. (c) Comparison of 95% confidence intervals of *v*_4_ determined by simulation of ^13^C-MFA using distinct measured metabolites.

Here, a computer simulation was performed to confirm the previous finding that a mixture of [U–^13^C]glucose and non-labeled glucose was better than 100% [1–^13^C]glucose as the carbon source to determine the flux in an anaplerotic reaction (Crown et al., 2015; Millard et al., 2014; Shupletsov et al., 2014) (“Example_4_Simulation.py”). For this purpose, an expanded toy model of the TCA cycle and the metabolic flux vector *v* shown in Fig. 4a were used (“Example_4_Simulation_model.txt”). Furthermore, it was assumed that the isotope labeling patterns of α-ketoglutarate (AKG), oxaloacetate (OAC), and phosphoenolpyruve (PEP) were observed via mass spectrometry.

For the case of ^13^C-MFA using 100% [1–^13^C]glucose as the carbon source (denoted as 100% [1–^13^C]glucose), the simulated MDV of AKG, OAC, and PEP was calculated using Eq. (1). From the simulated MDV, an artificial observed MDV was generated by the addition of Gaussian noise (σ = 0.01; see Materials and Methods for the detailed procedure). Subsequently, a ^13^C-MFA was simulated by identifying an optimum metabolic flux vector using the artificial observed MDV data and determining the 95% confidence intervals of three anaplerotic reactions (*v*_4_, *v*_5_, and *v*_6_) and one non-anaplerotic reaction (*v*_7_). The procedure was repeated 100 times to yield a population of 95% confidence intervals, because the results of the ^13^C-MFA simulation depend on Gaussian noise used to create the artificial observed MDV. The median 95% confidence interval was used as a representative value.

Fig. 4b shows that the width of the 95% confidence interval of *v*_4_ (PEP carboxylase reaction) was 68 (range, 48–116). The 95% confidence interval was five times larger than that of *v*_7_ (pyruvate dehydrogenase reaction), with a 95% confidence interval of 15 (range, 98–113). Similar wide confidence intervals were observed for other anaplerotic reactions (*v*_5_ and *v*_6_). Moreover, an identical procedure was performed using another carbon source containing [U–^13^C]glucose and non-labeled glucose at a 50:50 ratio (denoted as 50% [U–^13^C]glucose). The width of the 95% confidence interval of reaction *v*_4_ was 36 (range, 50–86). The metabolic network (Fig. 4a) shows that reactions *v*_4_ and *v*_5_ form a reversible interconversion that mixes the ^13^C-labeling patterns of PEP and OAC. This means that an estimation of the interconversion level will be difficult when the overall percentage of ^13^C in PEP and OAC is low. Because the overall percentage of ^13^C in 100% [1–^13^C]glucose (17%) is lower than that of 50% [U–^13^C]glucose (50%), these results suggest that 100% [1–^13^C]glucose is an undesirable carbon source compared to 50% [U–^13^C]glucose for the analysis of the anaplerotic reactions.

To reduce the range of the confidence interval, a mixture of [1–^13^C]glucose:[U–^13^C]glucose at 50:50 is likely a better carbon source because of interactions between [1–^13^C]glucose and [U–^13^C]glucose (denoted as Mixture). Alternatively, it has been reported that the parallel execution of multiple labeling experiments, such as two labeling experiments using 100% [1–^13^C]glucose and 50% [U–^13^C]glucose, can improve the flux estimation performance (Parallel) (Ahn and Antoniewicz, 2013; Leighty and Antoniewicz, 2013; Maeda et al., 2016). The computer simulation of the Mixture and Parallel cases showed that the 95% confidence intervals determined by the Mixture were similar to that of 100% [1–^13^C]glucose, indicating that no specific interaction occurred in the mixing of the carbon source to decrease the intervals. In contrast, the results supported that the parallel labeling experiment was useful for improving the flux estimation because the 95% confidence intervals of all reactions were the narrowest among the four carbon sources.

Next, based on the experimental design, we determined the more important metabolite or mass isotopomer for estimating metabolic flux levels. As mentioned above, it was assumed that the isotope labeling patterns of AKG, OAC, and PEP were observed via mass spectrometry in the computer simulation of ^13^C-MFA. One approach to identify better target metabolites for measurement is to compare the simulated 95% confidence intervals using distinct sets of target metabolites. As previously stated, the 95% confidence interval of *v*_4_ was determined to be 36 (from 50 to 86) for the computer simulation of ^13^C-MFA using 50% [U–^13^C]glucose as the carbon source (Fig. 4b and c). Additional computer simulations showed that the 95% confidence intervals expanded to 62 (AKG and OAC), 129 (AKG and PEP), and 43 (OAC and PEP) when the isotope labeling patterns of the two metabolites were used for the ^13^C-MFA (Fig. 4c). The results showed that the MDV data of OAC were more important than those of other metabolites because the 95% confidence intervals expanded significantly when the MDV data of OAC were removed from the MDV dataset. These examples show that mfapy can describe various data analysis workflows for the computer simulation of ^13^C-based metabolic flux analysis.

### 3.6 Function of mfapy: computational basis for developing alternative methodologies

The flexibility and extensibility provided by mfapy can support the development of new data analysis techniques for stable isotope labeling experiments, such as a Bayesian approach (Heinonen et al., 2019; Kadirkamanathan et al., 2006). In this study, a Markov chain Monte Carlo (MCMC) algorithm was applied to select useful mass isotopomers for ^13^C-MFA. From a Bayesian perspective, we did not possess any information regarding the metabolic flux vector *v* or the metabolic flux levels of *v* before measuring the MDV of the target metabolites. After measuring the MDV of the target metabolites, we estimated the posterior distribution of *v* under the condition that the MDV data were observed. The probability distribution of flux vector *v*, *P* (RSS(*v*)), should follow a χ^2^ distribution (degree of freedom is the number of measurements) because the observed MDV data include an experimental error following the normal distribution. The Metropolis–Hastings method is an MCMC algorithm that generates a posterior distribution of *v* (see Materials and Methods for the detailed procedure).

**Fig. 5:**
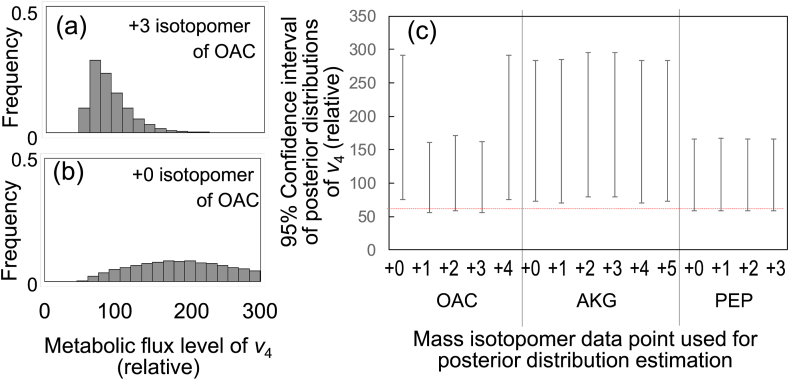
A Bayesian approach to determine important metabolites or mass isotopomers for estimating metabolic flux levels. Computer simulations were performed using the expanded toy model for the case of 50% [U–^13^C]glucose as the carbon source. (a and b) Posterior distributions of metabolic flux level of *v*_4_ determined using Metropolis–Hastings method when +3 isotopomer (a) and +0 isotopomer (b) of OAC were used as the measured data. (c) Comparison of 95% intervals of posterior distributions when each isotopomer, i.e., OAC, AKG, and PEP, was used as the measured data. In all simulations, [U–^13^C]glucose and non-labeled glucose at a 50:50 ratio were used as carbon sources.

An interesting application of this approach is the generation of a posterior distribution from a small number of MDV data points. A posterior distribution can be generated even if the number of data points is less than the degree of freedom of the metabolic model. Using the expanded toy model of the TCA cycle, we prepared a simulated MDV using the procedure described above without the addition of Gaussian noise. The relative intensity of the +3 isotopomer of the OAC was obtained from the simulated MDV. Using only one data point, a posterior distribution of *v*_4_ was successfully obtained using the Metropolis–Hastings method, as shown in Fig. 5a. The metabolic flux levels of the 2.5 and 97.5 percentile points of the posterior distribution were 55 and 161, respectively. In contrast, the same analysis using the +0 isotopomer of OAC yielded an extremely broad distribution (Fig. 5b). These results suggest that the observation of the +3 isotopomer of OAC was more critical than that of the +0 isotopomer of OAC for estimating the metabolic flux level of *v*_4_. Fig. 5c shows the metabolic flux levels of 2.5 and 97.5 percentile points of the posterior distributions determined for every isotopomer of OAC, AKG, and PEP. The comparison showed that the +1, +2, and +3 isotopomers of OAC were the most important measurement targets for estimating the metabolic flux level of *v*_4_, and that the result was identical to that of the 95% confidence interval-based method (Fig. 4c).

To implement the Metropolis–Hastings method, we first developed an mfapy-based Python code describing the procedure (“Example_5_1_MonteCalro.py”). Subsequently, the method to execute the entire Metropolis–Hastings algorism was newly added to a MetabolicModel class (“Example_5_2_MonteCalro.py”). A generation of one posterior distribution of *v* required approximately 0.5 h, whereas the determination of the 95% confidence intervals of 17 flux levels in the previous section required 4.3 h (using AMD Ryzen 9 3900 × 12 core processor). The demonstration suggested that extendability as an open-source Python package can provide a computational basis for developing alternative methodologies for ^13^C-labeling experiments.

### 3.7 Function of mfapy: isotopically non-stationary (INST)-MFA

mfapy supports INST-MFAs (Jazmin et al., 2014). The INST-MFA additionally needs pool size data of intracellular metabolites and time-course MDV data. The example python code, “Example_6_INSTMFA_toymodel.py,” describes a procedure for a computer simulation of INST-MFA using the toy model. The pool size levels of each metabolite can be considered as “free,” “fixed,” and “fitting” similar to the metabolite flux levels. By setting the time point information, the generate_mdv method can generate the MdvTimeCourseData class to deal with a time-course MDV dataset (detailed information is available from the mfapy documentation web page, https://fumiomatsuda.github.io/mfapy-document/).

In our previous study, INST-MFA was applied to *Synechocystis* sp. PCC 6803 GT strain under photoautotrophic conditions using NaH^13^CO_3_ as the carbon source (Nakajima et al., 2017). Using the time course MDV data for 15 fragments of 13 free metabolites, an optimal metabolic flux distribution and metabolite pool size, which passed the χ^2^ test with α = 0.05, were estimated using non-linear fitting of predicted and measured time courses of MDV in the previous study (Nakajima et al., 2017). However, the fitting took an enormous amount of time (approximately 1 week) because numerical integration is needed to calculate the simulated time-course MDV data during the non-linear fitting.

## 4 Discussion

The Python package mfapy was developed to provide a toolbox for ^13^C-MFA. mfapy compels users to write Python codes and has no user-friendly graphical interface. However, various functions, including the availability of multiple solvers (Fig. 2), parallel execution of the optimization task (Fig. 3), generation of artificially observed MDV for simulating ^13^C-MFA experiments (Fig. 4), the basis for developing alternative methodologies (Fig. 5), and INST-MFA can facilitate ^13^C-MFA studies.

The availability of multiple solvers and the parallel execution of optimization tasks of mfapy enabled trial and error to obtain the global optimum, which have contributed to the success of previous ^13^C-MFA studies of *E. coli* (Wada et al., 2017), yeast (Hayakawa et al., 2015), and cultured breast cancer cells (Araki et al., 2018; Okahashi et al., 2015, Okahashi et al., 2018). Moreover, a better design for various ^13^C-MFA experiments was obtained by computer simulation of the ^13^C-labeling experiment. For instance, a computer simulation using mfapy revealed that 100% [2–^13^C]ethanol was the best carbon source among [1–^13^C]ethanol and [U–^13^C]ethanol for the ^13^C-labeling experiment of ethanol-assimilating *Saccharomyces cerevisiae* (budding yeast) (Hayakawa et al., 2018).

Furthermore, the flexibility of mfapy provided a basis for new data analysis techniques, including a Monte Carlo-based data analysis method recently developed for the quantitative assessment of metabolic reprogramming (Matsuda et al., 2020) (Example_5_2_MonteCalro.py). Flux analysis or isotopomer analysis instead of ^13^C-MFA has been widely used to investigate metabolic redirection and reprogramming, such as in cancer and immune cells (Brekke et al., 2012; Dong et al., 2017; Fan et al., 2014; Faubert et al., 2017; Lewis et al., 2014; Liu et al., 2016; Lussey-Lepoutre et al., 2015). In many cases, the metabolic flux ratio between the two pathways has been estimated from the ^13^C-labeling patterns or MDV of a few metabolites. For such cases, a Monte Carlo-based approach would be useful because it can generate a probability distribution of the metabolic flux vector *v* using a small number of data points. The deliverables were newly implemented in the mfapy package in this study.

mfapy has been continuously developed to enrich functions, such as loading various model definition formats and data visualization (Beyss et al., 2019). New versions will be available to the public from the Github repository.

## CRediT author statement

Fumio Matsuda: Methodology, Software, Investigation, Writing – original draft, Reviewing, and Editing, Kousuke Maeda: Software, and Investigation, Takeo Taniguchi: Software, and Investigation, Yuya Kondo: Software, and Investigation, Futa Yatabe: Software, and Investigation, Nobuyuki Okahashi:, Methodology, Software, Investigation, Reviewing, and Editing, and Hiroshi Shimizu: Conceptualization and Supervision.

## Research data

The Python script and model definition file can be downloaded from our GitHub repository: https://github.com/fumiomatsuda/mfapy.

## Declaration of competing interest

The authors declare that they have no known competing financial interests or personal relationships that could have appeared to influence the work reported in this paper.

## Appendix List of abbreviations

MDV: mass isotope distribution vector
^13^C-MFA: ^13^C-based metabolic flux analysis
INST-^13^C-MFA: isotopically nonstationary ^13^C-metabolic flux analysis
AKG: α-ketoglutarate
AcCoA: acetyl-CoA
Cit: citrate
F6P: fructose-6-phosphate
FBP: fructose-1,6-bisphosphate
FUM: fumarate
G6P: glucose-6-phosphate
GAP: glyceraldehyde-3-phosphate
Gly: glycine;
OAC: oxaloacetate
Phe: phenylalanine;
PEP: phosphoenolpyruvate
3 PG: 3-phosphoglycerate
Pyr: pyruvate; SUC: succinate
