# Supplementary material for: mfapy: An open-source Python package for 13C-based metabolic flux analysis
Source: Metab Eng Commun. 2021 Jul 17;13:e00177. doi: 10.1016/j.mec.2021.e00177 (PMC8322459; doi:10.1016/j.mec.2021.e00177)
Supplement: Multimedia component 1 [file mmc1.pdf]

Supplementary Information

mfapy: An open-source Python package for ^{13}C-based metabolic flux analysis

Fumio Matsuda, Kohsuke Maeda, Takeo Taniguchi, Yuya Kondo, Futa Yatabe, Nobuyuki Okahashi, and Hiroshi Shimizu

Supplementary Figure 1 Example python script for ^{13}C-MFA of toy model using mfapy.
Supplementary Figure 2 Toy model of TCA cycle obtained from original article of EMU algorism.
Supplementary Figure 3 Metabolic model for ^{13}C-MFA of metabolically engineered *E. coli* considering G-value parameter.
Supplementary Figure 4 Functional test of mfapy

Supplementary Table 1 Model definition file of toy model (Example_1_toymodel_model.txt)
Supplementary Table 2 Calculation of mass isotope distribution vector (MDV) of glutamate by elementary metabolite unit (EMU) framework.
Supplementary Table 3 Comparison of results of ^{13}C-metabolic flux analysis of metabolically engineered *E. coli*.
Supplementary Table 4 Comparison between observed MDVs of glycine and phenylalanine; simulated MDVs of global and local optimums of ^{13}C-metabolic flux analysis of metabolically engineered *E. coli*.

Supplementary Figure 1 Example python script for ^{13}C-MFA of toy model using mfapy. Python codes for parallel labeling experiment are shown. (a) Following the import of mfapy package, (b) a metabolic model (model) is constructed from a model definition file using information regarding a metabolic network (reactions), reversible reactions (reversible), all metabolites in the metabolic network (“metabolites”), and the fragment of target metabolites whose labeling patterns are observed using mass spectrometry (“fragments”). Experimental conditions were configured by (c) addition of constraints to metabolic flux vector, (d) preparation of two ^{13}C-labeled carbon sources, and (e) loading two MDV datasets for parallel labeling experiment. (f) Metabolic flux distribution was estimated by setting experiments, generating random initial flux vectors, and non-linear optimization. (g) Lower and upper boundaries of 95% confidence interval of v3 were estimated as an example.

```
#---------------------------------------------------------------------------------
# Name:        mfapy Example_1_toymodel.py Example code of mfapy
#---------------------------------------------------------------------------------
import mfapy                                                                     (a)
# Model construction
reactions, reversible, metabolites, fragments¥
 = mfapy.mfapyio.load_metabolic_model("example_1_toymodel_model.txt")           (b)
model = mfapy.metabolicmodel.MetabolicModel(reactions, reversible,¥
 metabolites, fragments)
# Addition of constraints
state = model.load_states("Example_1_toymodel_status.csv", format = 'csv')
model.set_constraints_from_state_dict(state)                                     (c)
model.update()
# Generation of CarbonSource instance
cs1 = model.generate_carbon_source_template()
cs1.set_each_isotopomer('AcCoA', {'#10':0.5})                                    (d)
cs2 = model.generate_carbon_source_template()
cs2.set_each_isotopomer('AcCoA', {'#11':0.5})
# Load MDV data
mdv1 = model.load_mdv_data("Example_1_MDV1.txt")                                 (e)
mdv2 = model.load_mdv_data("Example_1_MDV2.txt")
# Flux estimation Step 1: Setting experimments
model.set_experiment('ex1', mdv1, cs1)                                           (f)
model.set_experiment('ex2', mdv2, cs2)
# Flux estimation step 2: Generation of intical flux vectors
endstate, flux = model.generate_initial_states(50, 4, method ="parallel")        (g)
# Flux estimation step 3: Fitting model
for method in ["GN_CRS2_LM", "LN_PRAXIS", "SLSQP"]:
    endstate, RSS, flux = model.fitting_flux(method = method, flux = flux)      (h)
model.show_results([("final", flux[0])]) # Show result
# Estimation of 95% CI
ci_edge = model.generate_ci_template(targets = [('reaction', "v3")])
ci = model.search_ci(ci_edge, flux[0], method = 'grid')
lb = ci['data'][('reaction', "v3")]['lower_boundary']                            (i)
ub = ci['data'][('reaction', "v3")]['upper_boundary']
print("v3", "Lower bondary:",lb, "Upper boundary:", ub)
```

Supplementary Figure 2 Toy model of TCA cycle obtained from original article of EMU algorism (Antoniewicz et al., 2007)

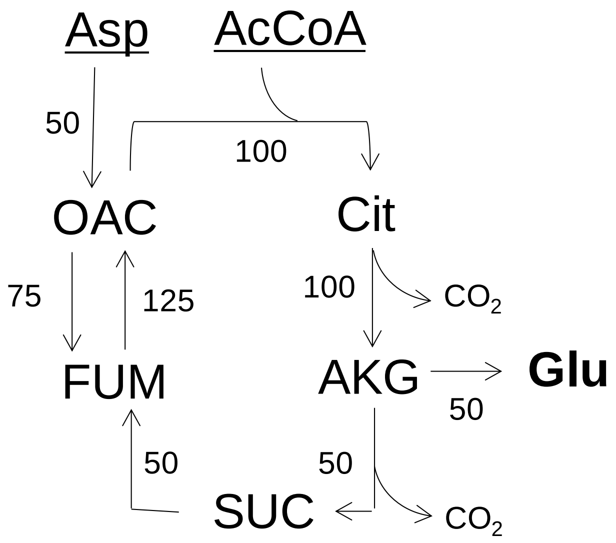

Supplementary Figure 3 Metabolic model for ^{13}C-MFA of metabolically engineered *E. coli* considering G-value parameter (extracted). Complete metabolic model is available as "Example_2_Ecoli_model.txt" from mfapy web page (https://github.com/fumiomatsuda/mfapy). G-value parameter is used to rectify effects of inoculated unlabeled proteinogenic amino acids (such as Ala in the network) on observed MDV. Hence, metabolic reaction from Pyr to Ala (r_{58}) was set as "pseudo." Because "pseudo"-type reactions were disregarded in constructing a stoichiometry matrix of its substrate, metabolic flux levels of r58 did not affect metabolic flux levels of Pyr-related reactions. However, metabolic flux levels of r_{58} and atom mapping was considered for its product. ^{13}C-labeling of Ala yielded was identical to that of Pyr. To represent effects of inoculated unlabeled proteinogenic Ala, the yielded Ala was mixed with non-labeled Ala (SubsAla) and then discarded as Alaex to maintain a metabolic steady state. Ratio between r_{58} and r_{95} in optimum flux vector was used to estimate G-value of Ala.

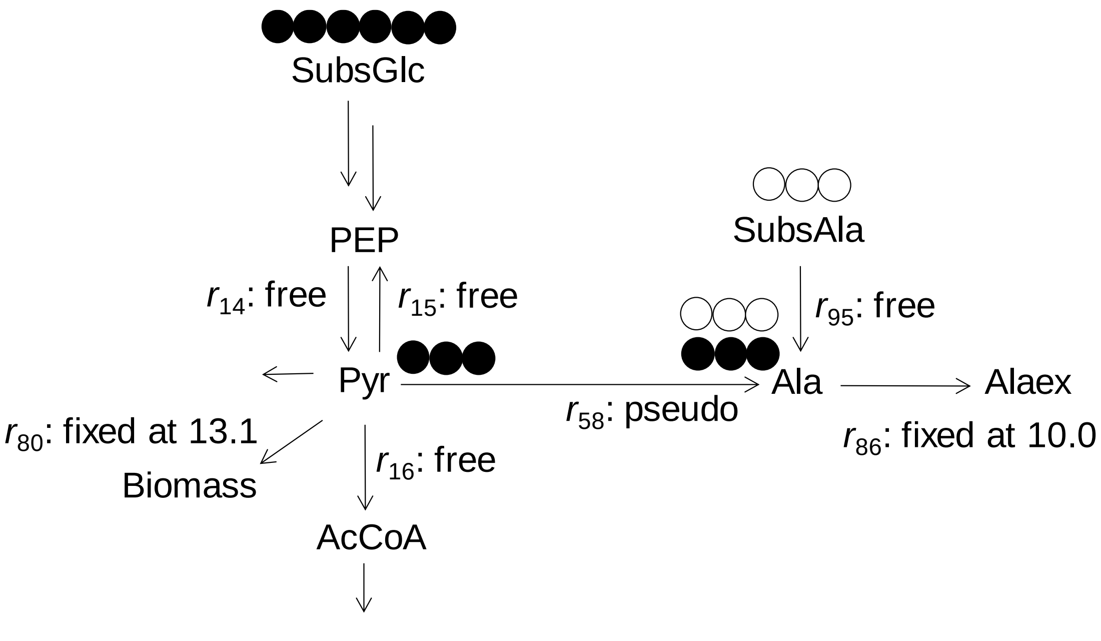

Supplementary Figure 4 Functional test of mfapy. Comparison of estimated metabolic flux vectors (v_{opt}) of ^{13}C-MFA of metabolically engineered *E. coli* determined using authentic software (OpenMebius) and mfapy. Metabolic models and measurement data were obtained from previous study (Okahashi et al., 2017).

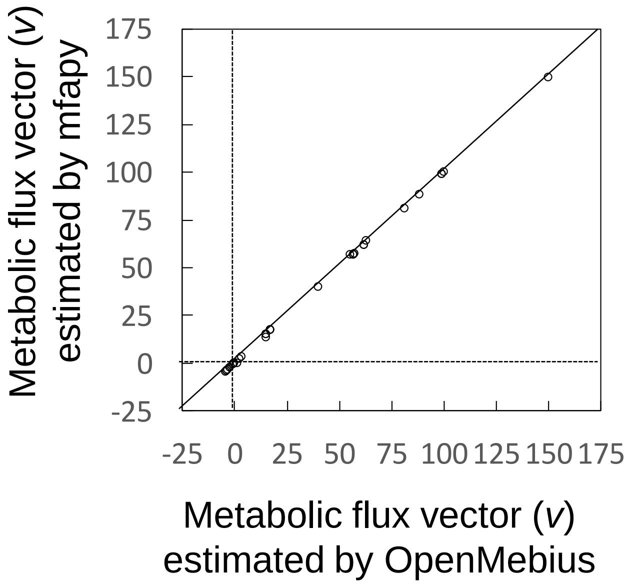

Supplementary Table 1 Model definition file of toy model (Example_1_toymodel_model.txt).

#					
# Toy model for example 1					
#					
//Reactions					
# ID	For stoichiometry matrix	For atom mapping	Atom mapping	External ID	
v1	AcCoA + OAC --> Cit	AcCoA + OAC --> Cit	AB + CDEF --> FEDBAC	(kegg:R00351)	
v2	Cit --> AKG + CO2ex	Cit --> AKG + CO2ex	ABCDEF --> ABCDE + F	(kegg:R00709)	
v3	AKG --> Glu	AKG --> Glu	ABCDE --> ABCDE	(kegg:R00243)	
v4	AKG --> Suc + CO2ex	AKG --> Suc + CO2ex	ABCDE --> BCDE + A	(kegg:R01197)	
v5	Suc --> Fum	Suc --> Fum	ABCD --> ABCD	(kegg:R02164)	
v6	Fum --> OAC	Fum --> OAC	ABCD --> ABCD	(kegg:R01082)	
v7	OAC --> Fum	OAC --> Fum	ABCD --> ABCD	(kegg:R01082)	
v8	Asp --> OAC	Asp --> OAC	ABCD --> ABCD	(kegg:R00355)	
v9	Glu --> Gluex	nd	nd	(kegg:R00243)	
//Metabolites					
# Name	Number of atom	Symmetry	Carbon source	Excreted metabolite	External ID
CO2ex	1	no	no	excreted	(kegg:C00011)
AcCoA	2	no	carbonsource	no	(kegg:C00024)
OAC	4	no	no	no	(kegg:C00036)
Cit	6	no	no	no	(kegg:C00158)
AKG	5	no	no	no	(kegg:C00026)
Suc	4	symmetry	no	no	(kegg:C00042)
Fum	4	symmetry	no	no	(kegg:C00122)
Glu	5	no	no	no	(kegg:C00025)
Gluex	5	no	no	excreted	(kegg:C00025)
Asp	4	no	carbonsource	no	(kegg:C00049)
//Reversible_reactions					
#Name	Forward reaction	Reverse reaction	External ID		
FUM	v6	v7	(kegg:R01082)		
//Target_fragments					
#Name	Type (gcms and msms)	Corresponding metabolite and atoms	Usage	Formula (Experimental)	
GluMes	gcms	Glu_1:2:3:4:5	use	C5H10N2O3	
//End					

Supplementary Table 2 Calculation of mass isotope distribution vector (MDV) of glutamate by elementary metabolite unit (EMU) framework.

Mass isotope distribution vector (MDV)	Calculated by mfapy	Theoretical value (Antoniewiez et al 2007)
Glutamate m+0	0.3464	0.3464
Glutamate m+1	0.2695	0.2695
Glutamate m+2	0.2708	0.2708
Glutamate m+3	0.0807	0.0807
Glutamate m+4	0.0286	0.0286
Glutamate m+5	0.0039	0.0039

Supplementary Table 3 Comparison of results of ^{13}C-metabolic flux analysis of metabolically engineered *E. coli*.

ID	Stoichiometry	Determined by mfapy (This study)	Determined by OpenMebius (Okahashi et al 2017)
r1	SubsGlc-->G6P	100.0	100
PGI	G6P<->F6P (net)	0.0	0
PFK	F6P<->FBP (net)	-3.7	-3.7
FBA	FBP<->DHAP+GAP (net)	-3.7	-3.7
TPI	DHAP<->GAP (net)	-4.3	-4.3
GAPDH	GAP<->3PG (net)	88.4	88.4
PEPH	3PG<->PEP (net)	81.1	81.2
PYK	PEP<->Pyr (net)	64.2	62.9
r16	Pyr-->AcCOA+CO2in	149.9	150
r17	AcCOA+Oxa-->IsoCit	61.9	61.8
r18	IsoCit-->aKG+CO2in	61.9	61.8
r19	aKG-->Suc+CO2in	56.8	56.7
SDH	r20<=>r21	56.8	56.7
FH	r22<=>r23	56.8	56.7
MDH	r24<=>r25	56.7	55.4
r26	IsoCit+AcCOA-->Mal+Suc	0.0	0
PEPK	PEP+CO2<->Oxa (net)	13.6	14.9
r42	Mal-->Pyr+CO2in	0.1	1.4
r27	G6P-->m6PG	99.0	99
r28	m6PG-->Ru5P+CO2in	0.0	0
RPI	r29<=>r30	3.4	3.4
RBE	r31<=>r32	-3.4	-3.4
TKT1	r33<=>r34	-0.8	-0.8
TAL	r35<=>r36	-0.8	-0.8
TKT2	r37<=>r38	-2.5	-2.5
r39	m6PG-->Pyr+GAP	99.0	99
r49	AcCOA-->Acetate	57.1	57.2
r50	Acetate-->Acetateex	40.0	40
r43	AcCOA+AcCOA-->AcAcCOA	17.1	17.2
r44	AcAcCOA+Acetate-->AcAc+AcCOA	17.1	17.2
r45	AcAc-->Acetone+CO2in	17.1	17.2
r46	Acetone-->IPA	15.0	15.1
r47	Acetone-->Acetoneex	2.1	2.1
r48	IPA-->IPAex	15.0	15.1

Supplementary Table 4 Comparison between observed MDVs of glycine and phenylalanine; simulated MDVs of global and local optimums of ^{13}C-metabolic flux analysis of metabolically engineered *E. coli*.

Fragment	Number of isotopes	observed MDV	Global optimum		Local optimum	
			Simulated MDV	RSS of Simulated MDV	Simulated MDV	RSS of Simulated MDV
Gly[M-57]	0	0.634	0.636	0.22	0.634	0.01
Gly[M-57]	1	0.105	0.106	0.01	0.107	0.08
Gly[M-57]	2	0.260	0.258	0.32	0.259	0.13
Gly[M-85]	0	0.720	0.718	0.32	0.720	0.00
Gly[M-85]	1	0.280	0.282	0.32	0.280	0.00
Phe[M-159]	0	0.233	0.234	0.06	0.236	0.51
Phe[M-159]	1	0.142	0.141	0.04	0.144	0.21
Phe[M-159]	2	0.193	0.194	0.01	0.195	0.16
Phe[M-159]	3	0.189	0.188	0.08	0.183	1.91
Phe[M-159]	4	0.096	0.096	0.01	0.096	0.01
Phe[M-159]	5	0.090	0.091	0.04	0.091	0.01
Phe[M-159]	6	0.036	0.036	0.01	0.035	0.08
Phe[M-159]	7	0.015	0.015	0.00	0.016	0.02
Phe[M-159]	8	0.006	0.006	0.01	0.005	0.04
